# Predictors of recurrence after total thyroidectomy in 1,611 patients with papillary thyroid carcinoma: postoperative stimulated serum thyroglobulin and ATA initial and dynamic risk assessment

**DOI:** 10.20945/2359-4292-2022-0506

**Published:** 2024-04-09

**Authors:** Andre Ywata de Carvalho, Hugo Fontan Kohler, Camila C. G. Ywata de Carvalho, Jose Guilherme Vartanian, Luiz Paulo Kowalski

**Affiliations:** 1 A.C. Camargo Cancer Center Departamento de Cirurgia de Cabeça e Pescoço e Otorrinolaringologia São Paulo SP Brasil A.C. Camargo Cancer Center, Departamento de Cirurgia de Cabeça e Pescoço e Otorrinolaringologia, São Paulo, SP, Brasil; 2 A.C. Camargo Cancer Center Divisão de Oncologia Cirúrgica São Paulo SP Brasil A.C. Camargo Cancer Center, Divisão de Oncologia Cirúrgica, São Paulo, SP, Brasil; 3 Universidade de São Paulo Departamento de Cirurgia de Cabeça e Pescoço Faculdade de Medicina São Paulo SP Brasil Faculdade de Medicina, Departamento de Cirurgia de Cabeça e Pescoço, Universidade de São Paulo, São Paulo, SP, Brasil

**Keywords:** Thyroid, papillary thyroid carcinoma, recurrence, risk assessment, thyroglobulin

## Abstract

**Objective:**

Despite a favorable prognosis, some patients with papillary thyroid carcinoma (PTC) develop recurrence. The objective of this study was to examine the impact of the combination of initial American Thyroid Association (ATA) risk stratification with serum level of postoperative stimulated thyroglobulin (s-Tg) in predicting recurrence in patients with PTC and compare the results with an assessment of response to initial therapy (dynamic risk stratification).

**Subjects and methods:**

We retrospectively analyzed 1,611 patients who had undergone total thyroidectomy for PTC, followed in most cases (87.3%) by radioactive iodine (RAI) administration. Clinicopathological features and s-Tg levels obtained 3 months postoperatively were evaluated. The patients were stratified according to ATA risk categories. Nonstimulated thyroglobulin levels and imaging studies obtained during the first year of follow-up were used to restage the patients based on response to initial therapy.

**Results:**

After a mean follow-up of 61.5 months (range 12-246 months), tumor recurrence was diagnosed in 99 (6.1%) patients. According to ATA risk, recurrence was identified in 2.3% of the low-risk, 9% of the intermediate-risk, and 25% of the high-risk patients (p < 0.001). Using a receiver operating characteristic curve approach, a postoperative s-Tg level of 10 ng/mL emerged as the ideal cutoff value, with positive and negative predictive values of 24% and 97.8%, respectively (p < 0.001). Patients with low to intermediate ATA risk with postoperative s-Tg levels < 10 ng/mL and excellent response to treatment had a very low recurrence rate (<0.8%). In contrast, higher recurrence rates were observed in intermediate-risk to high-risk patients with postoperative s-Tg ≥ 10 ng/mL and indeterminate response (25%) and in those with incomplete response regardless of ATA category or postoperative s-Tg value (38.5-87.5%). Using proportion of variance explained (PVE), the predicted recurrence using the ATA initial risk assessment alone was 12.7% and increased to 29.9% when postoperative s-Tg was added to the logistic regression model and 49.1% with dynamic risk stratification.

**Conclusions:**

The combination of ATA staging system and postoperative s-Tg can better predict the risk of PTC recurrence. Initial risk estimates can be refined based on dynamic risk assessment following response to therapy, thus providing a useful guide for follow-up recommendations.

## INTRODUCTION

Papillary thyroid carcinoma (PTC) has been increasing in incidence worldwide (1). Excellent outcomes have been demonstrated with PTC therapy, with 10-year survival rates of 93% (2), but up to 28% of the patients develop locoregional recurrence (3).

The risk of PTC recurrence can be estimated from clinicopathological features like age, presence of extrathyroidal extension, or regional metastases (4). Measurement of serum thyroglobulin (Tg) levels after TSH stimulation (stimulated Tg [s-Tg]) may help assess disease persistence or presence of thyroid remnant and predict potential cancer recurrence (5). Indeed, high s-Tg levels after total thyroidectomy can predict PTC recurrence (6). The 2015 ATA guidelines for the management of thyroid cancer propose a system to estimate the risk of PTC relapse based on clinicopathological findings (7). While this risk stratification system provides a valuable tool in predicting recurrence risk, additional information obtained during follow-up can effectively refine the initial risk estimate by incorporating response to therapy variables (suppressed Tg levels and imaging studies) (8). According to the ATA guidelines, patients with postoperative serum Tg suggestive of distant metastases are classified as being at high risk for recurrence. However, no consensus has been established on the usefulness of postoperative s-Tg or the optimal cutoff value in predicting disease severity or therapeutic response (9,10). Finally, no studies to date have assessed the impact of treatment response on initial estimates of recurrence risk when postoperative s-Tg is incorporated into the ATA risk classification.

Based on the above, the aim of this study was to review the characteristics of patients with PTC who underwent total thyroidectomy followed or not by RAI administration and to examine their risk of recurrence based on clinicopathological characteristics at diagnosis, ATA risk stratification, and postoperative s-Tg level. Another aim of the study was to evaluate the impact of assessing the response to initial therapy on modifying these initial risk estimates.

## SUBJECTS AND METHODS

### Study population and treatment

**Figure 1:**
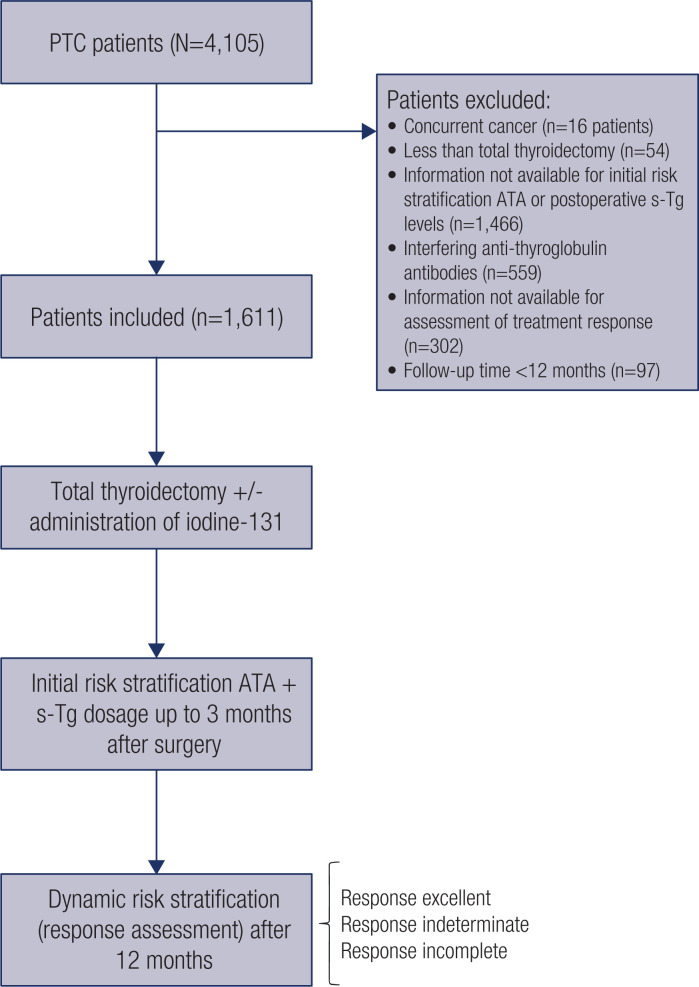
Participant flowchart. PTC: papillary thyroid carcinoma; ATA: American Thyroid Association; s-Tg: stimulated thyroglobulin.

After institutional review board approval (number 3,055/21; Plataforma Brasil number 43466521.8.0000.5432), we retrospectively reviewed the medical records of patients who underwent thyroidectomy due to PTC between January 1996 and December 2015. The study included 1,611 patients who met all of the following criteria: a postoperative diagnosis of PTC, total thyroidectomy or subsequent completion thyroidectomy, s-Tg levels obtained up to 3 months postoperatively, and a minimum follow-up of 1 year, unless recurrence or death occurred before this time point. We excluded patients with concurrent thyroidal malignancies, interfering antithyroglobulin antibodies, and insufficient information for initial staging and risk restratification (Figure 1). Patients with pathologically confirmed high-risk findings (mainly extrathyroidal extension and lymph node metastasis) received routine RAI treatment. Patients categorized as low risk had s-Tg levels measured in the first 3 months after total thyroidectomy to determine the extent of remnant normal thyroid tissue or presence of subclinical disease, with some receiving RAI ablation. Serum levels of TSH, s-Tg, and antithyroglobulin antibodies were measured before RAI administration, and whole-body RAI scans were performed 2-5 days after RAI therapy. Serum Tg levels were determined using different immunoassays with functional sensitivities of 0.1-0.2 ng/mL (the most common immunoassay was Immulite 2000, Siemens Healthcare Diagnostics Inc., Deerfield, IL, USA). Serum levels of antithyroglobulin antibodies were measured by radioimmunoassay (normal values < 60 IU/L; Brahms GmbH, Hennigsdorf, Germany). Follow-up visits included palpation of the neck, neck ultrasound, and measurement of serum levels of TSH, Tg, and antithyroglobulin antibodies.

### Prognostic parameters

The patients’ characteristics, therapeutic information, pathological features, and postoperative clinical outcomes were analyzed. The patients were classified according to the 2015 ATA risk stratification system as having a low, intermediate, or high risk for recurrence (7) and were further stratified by postoperative s-Tg levels (11–15). Finally, patients were divided into three groups according to the 2015 ATA dynamic risk stratification, based on laboratory and imaging findings during the first year of follow-up: excellent response (nonstimulated Tg [ns-Tg] ≤ 0.2 ng/mL after total thyroidectomy + RAI or ns-Tg ≤ 1 ng/mL after total thyroidectomy without RAI, no rising levels of antithyroglobulin antibodies and normal postoperative neck ultrasound); incomplete response (ns-Tg ≥ 1 ng/mL after total thyroidectomy + RAI or ns-Tg ≥ 5 ng/mL after total thyroidectomy without RAI, rising antithyroglobulin antibody levels, persistent or newly identified locoregional or distant metastases), and indeterminate response (nonspecific biochemical or structural findings, which could not be confidently classified as either benign or malignant) (16). Structural disease recurrence was defined as the reappearance of cancer after the initial therapy and included all events reported (local recurrences, lymph node metastases, and distant metastases) and confirmed by biopsy/surgery.

### Statistical analysis

The primary endpoint of the study was the recurrence-free survival (RFS). The RFS probabilities were estimated using the Kaplan-Meier method, and differences among the survival curves of each variable were verified using the log-rank test. Crude and multivariate hazard ratios of recurrence, along with their respective 95% confidence intervals (95% CIs) were estimated using Cox proportional hazards model. The backward selection technique with a p value < 0.10 was used to select variables for the final model. Schoenfeld and scaled Schoenfeld residuals were calculated by checking whether proportional hazards assumption would hold for the final multivariate Cox regression model. A receiver operating characteristic (ROC) curve approach was used to identify the postoperative s-Tg level of optimal test sensitivity and specificity. We used the proportion of variance explained (PVE), estimated from a logistic regression model of recurrence using the Nagelkerke/Cragg-Uhler test (17), to compare the predictive capacity of recurrence of the following variables: ATA risk category and postoperative s-Tg, alone or in combination, and the pattern of response to treatment. The statistical analysis was performed using Stata, version 16 (StataCorp LP, College Station, Texas, USA) and R (www.cran.org).

## RESULTS

The clinicopathological characteristics, risk stratification, treatment-related variables, postoperative s-Tg levels, and clinical outcomes of the cohort are presented in Table 1.

**Table 1:** Characteristics of the cohort

Characteristics			N patients
Sex	Female	76.1%	1,226
Age (years):	Mean (range)	43.7 (7-83)	1,611
Tumor size (mm):	Mean (range)	12.8 (0.4-140)	1,611
	≤10 mm	54.7%	881
Aggressive histology		4.3%	70
Multifocality		40.3%	649
Bilaterality		29.2%	471
Extrathyroidal extension	No	72.9%	1,174
	Minor	20.7%	334
	Gross	6.4%	103
Lymphovascular invasion		2%	33
Lymph node metastasis	cN1	12.8%	206
	pN0	3.6%	58
	pN1a	15.9%	256
	pN1b	6.3%	102
Chronic lymphocytic thyroiditis		20.8%	335
ATA risk stratification category	Low	57.8%	932
	Intermediate	35.7%	575
	High	6.5%	104
Neck lymph node dissection	No	79.7%	1,284
	Central (level VI)	13.8%	223
	Central plus lateral (II-VI)	6.5%	104
RAI ablation	Yes	87.3%	1,407
	Mean (range)	135 (30-389.5)	
Postoperative s-Tg	Mean (SD)	27.3 (368.4)	
	Median (range)	2.5 (<0.2-10,333)	
	≤1 ng/mL	30.4%	489
	1-10 ng/mL	51.1%	823
	≥10 ng/mL	18.5%	299
Clinical response to initial therapy classification	Complete	80.1%	1,291
	Biochemical incomplete	4%	64
	Structural incomplete	2.7%	43
	Indeterminate	13.2%	213
Follow-up (years)	Mean (range)	61.5 (12-246)	
Recurrence	Total	6.1%	99
	During the first 2 years	60.6%	60
	3-5 years	28.3%	28
	After 5 years	11.1%	11

Abbreviations: ATA: American Thyroid Association; RAI: radioiodine; SD: standard deviation; s-Tg: stimulated thyroglobulin.

### Correlation between ATA risk ± postoperative stimulated thyroglobulin and response to treatment

Patients with ATA high-risk PTC had a higher median postoperative s-Tg (6.2 ng/mL) than those with intermediate-risk (2.9 ng/mL) and low-risk (2.1 ng/mL; p < 0.001) PTC. Likewise, the median postoperative s-Tg was higher in patients with an incomplete response to treatment compared with those with an indeterminate or excellent response (23.0 ng/mL, 6.4 ng/mL, and 2.0 ng/mL, respectively; p < 0.001). The ATA risk category and postoperative s-Tg correlated strongly with response to initial therapy. For example, an excellent response to initial treatment was most common in patients classified as low risk (86.5%) or with postoperative s-Tg < 10 ng/mL (87.9%) compared with those with intermediate and high risk (54.8%-74.4%) or with higher s-Tg levels (46.2%) (Figure 2A). When these two variables were combined, patients with postoperative s-Tg < 10 ng/mL were more likely to achieve an excellent response, regardless of ATA risk classification, when compared with patients with higher s-Tg levels (76.2%-90.3% *versus* 22%-59.8%, respectively) (Figure 2B).

**Figure 2A:**
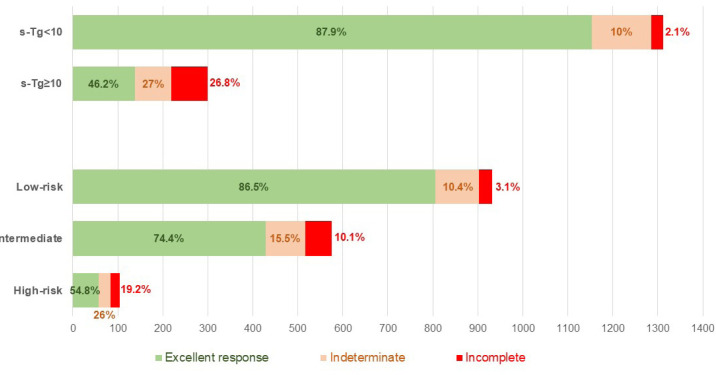
Response after initial therapy according to American Thyroid Association risk category and postoperative stimulated thyroglobulin (sTg) level in patients with papillary thyroid carcinoma.

**Figure 2B:**
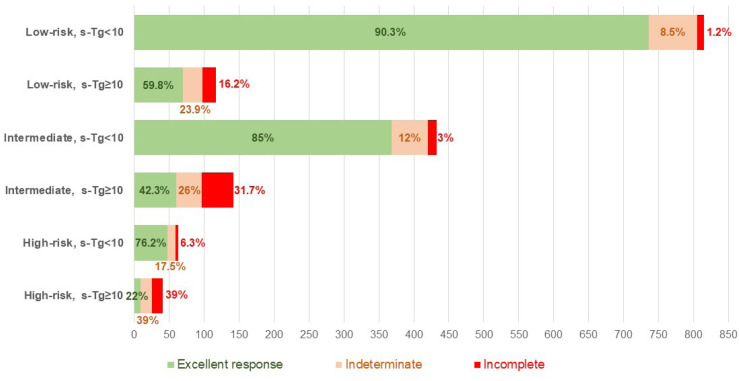
Response after initial therapy according to the combination of American Thyroid Association risk category and postoperative stimulated thyroglobulin (sTg) level in patients with papillary thyroid carcinoma.

### Recurrence

After a mean follow-up of 61.5 months (range 12-246 months), tumor recurrence was diagnosed in 99 patients (6.1%). In these patients, regional and distant recurrences were seen in 92 (92.9%) and 10 (10.1%), respectively. Most recurrences (60.6%) were diagnosed within the first 2 years of follow-up.

### Predictors of recurrence

**Figure 3:**
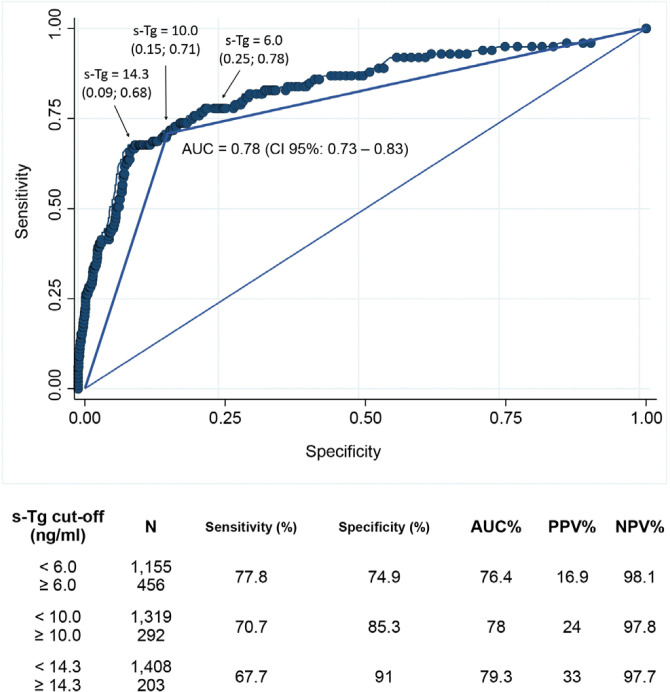
Time-dependent receiver operating characteristic curve for the prediction of disease-free survival using postoperative stimulated thyroglobulin (s-Tg) in patients treated for papillary thyroid carcinoma. AUC: area under the curve: PPV: positive predictive value: NPV: negative predictive value.

On Cox multivariate regression analysis, cancer recurrence was independently associated with ATA intermediate or high risk, as well as postoperative s-Tg ≥ 10 ng/mL (Table 2). Using the ROC curve approach, different serum postoperative s-Tg levels were analyzed for recurrence risk assessment and are shown in Figure 3. A postoperative s-Tg level of 10 ng/mL was identified as the ideal cutoff point, with a sensitivity of 70.7%, specificity of 85.3%, positive predictive value of 24%, and negative predictive value of 97.8% (area under the curve 0.78, 95% CI 0.73-0.83, p < 0.001).

**Table 2:** Univariate and multivariate logistic analyses of risk of recurrence in patients with papillary thyroid carcinoma

		Univariate Analysis		Multivariate Analysis	
		HR (95% CI)*	P value**	HR (95% CI)*	P value**
Male sex		1.81 (1.20-2.75)	0.005	1.23 (0.80-1.91)	0.347
Age < 55 years		0.66 (0.37-1.16)	0.146		
Tumor size > 10 mm		2.20 (1.47-3.31)	<0.001	1.09 (0.69-1.71)	0.715
Multifocality		1.14 (0.76-1.70)	0.526		
Bilaterality		1.49 (0.99-2.24)	0.057		
Chronic thyroiditis		0.64 (0.38-1.07)	0.089		
ATA risk category	Low	1		1	
	Intermediate	5.19 (3.12-8.63)	<0.001	3.07 (1.81-5.19)	<0.001
	High	11.01 (6.18-19.16)	<0.001	4.46 (2.32-8.57)	<0.001
Postoperative s-Tg level (ng/mL)	<1	1		1	
	1-10	2.10 (0.89-4.95)	0.089	1.99 (0.83-4.74)	0.121
	≥10	16.25 (7.47-35.32)	<0.001	10.67 (4.8-23.6)	<0.001

Abbreviations: ATA: American Thyroid Association; 95% CI: 95% confidence interval; HR: hazard ratio; s-Tg: stimulated thyroglobulin.

* HR and 95% CI estimated by Cox regression models.

** P value calculated using the log-rank test.

### Incorporation of postoperative stimulated thyroglobulin into ATA risk analysis for recurrence

**Figure 4:**
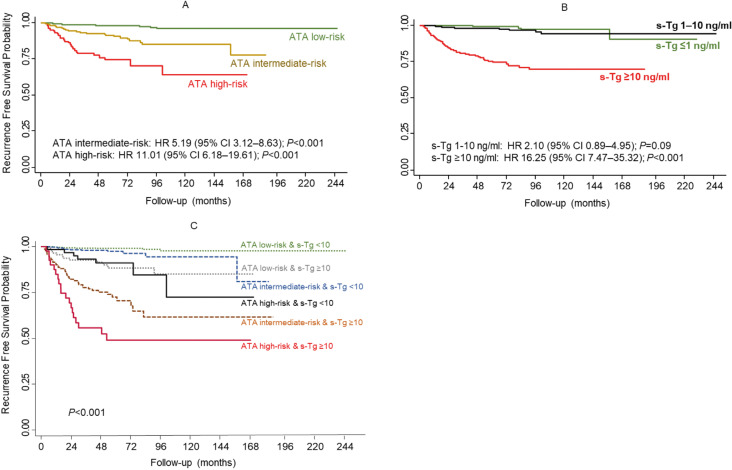
Influence of American Thyroid Association (ATA) initial risk stratification and postoperative stimulated thyroglobulin (s-Tg) level on the probability of recurrence-free survival in patients treated for papillary thyroid carcinoma. **(A)** Kaplan Meier survival based on ATA initial risk stratification. **(B)** Kaplan Meier survival based on s-Tg level. Note that the difference in recurrence risk is not statistically significant between patients with s-Tg ≤ 1 and 1-10 ng/mL (P=0.09). **(C)** Kaplan Meier survival according to the combination of ATA initial risk stratification and s-Tg (< or ≥ 10 ng/mL).

The 5-year RFS probability was significantly lower in patients categorized as high risk (73.8%) than in those categorized as intermediate risk (89.5%) and low risk (97.7%) according to the ATA risk category (Table 3 and Figure 4A). Similarly, a postoperative s-Tg ≥ 10 ng/mL was associated with a lower 5-year RFS probability (74.3%) compared with levels < 10 ng/mL (97.9%). The risk of recurrence was not significantly different between patients with s-Tg ≤ 1 ng/mL and 1-10 ng/mL (p = 0.09) (Figure 4B). When incorporated into the ATA risk analysis, postoperative s-Tg significantly altered the chance of tumor recurrence in all categories (Table 3 and Figure 4C). For example, patients with low to intermediate risk and low postoperative s-Tg (<10 ng/mL), who represented 77.5% of our cohort, had an excellent prognosis, with a 5-year RFS probability greater than 96.4%. In contrast, patients with intermediate to high risk and postoperative s-Tg ≥ 10 ng/mL had a 5-year RFS probability below 70.4%.

**Table 3:** Probability of recurrence-free survival according to the American Thyroid Association (ATA) risk stratification system and postoperative stimulated thyroglobulin level in patients treated for papillary thyroid carcinoma

		n (%)	5-year RFS %	Univariate Analysis	
				HR* (95% CI)	P value**
	Total	1,611 (100)	93.4		
ATA risk	Low	932 (57.8)	97.7	1.0 (ref.)	
	Intermediate	575 (35.7)	89.5	4.46 (2.68-7.41)	<0.001
	High	104 (6.5)	73.8	12.74 (7.16-22.65)	<0.001
Postoperative s-Tg (ng/mL)	<10	1,311 (81.4)	97.9	1.0 (ref.)	
	≥10	300 (18.6)	74.3	12.24 (7.90-18.96)	<0.001
ATA risk and s-Tg (ng/mL)	Low/<10	815 (50.6)	99.1	1.0 (ref.)	
	Low/≥10	117 (7.3)	88.5	9.79 (4.13-23.25)	<0.001
	Intermediate/<10	433 (26.9)	96.4	2.77 (1.17-6.58)	0.021
	Intermediate/≥10	142 (8.8)	70.4	29.62 (14.36-61.08)	<0.001
	High/<10	63 (3.9)	91.2	10.6 (3.95-28.47)	<0.001
	High/≥10	41 (2.5)	48.0	57.29 (25.88-126.8)	<0.001

Abbreviations: ATA: American Thyroid Association; 95% CI: 95% confidence interval; HR: hazard ratio; s-Tg: postoperative stimulated thyroglobulin; RFS: recurrence-free survival; ref.: reference.

* HR and 95% CI estimated by Cox regression models.

** P value calculated using the log-rank test.

### Impact of dynamic risk stratification

The data in Table 4 show the impact of the treatment response pattern on modifying the initial estimates of cancer recurrence based on ATA risk category and postoperative s-Tg level. Patients who demonstrated an excellent response to treatment had a good prognosis, with a low probability of relapse, especially those with low-risk to intermediate-risk and/or s-Tg levels < 10 ng/mL (0.3%-1.2%). On the other hand, patients with an incomplete response to initial therapy were more likely to have disease recurrence (38.5%-87.5%). The impact of dynamic risk stratification in modifying initial risk estimates was evident. An excellent response to therapy resulted in a significant decrease in the likelihood of thyroid cancer recurrence: from 25% to 5.3% in high-risk patients and from 23.7% to 3.6% in patients with postoperative s-Tg ≥ 10 ng/mL. In contrast, an incomplete response to therapy was associated with an increased likelihood of recurrence in each of the initial risk assessment groups: 2.3%-48.3% in low-risk patients, 9–58.6% in intermediate-risk patients, and 2.1%-51.9% in patients with postoperative s-Tg < 10 ng/mL. Finally, we compared the ability to predict recurrence using the initial ATA risk score and the postoperative s-Tg, alone or in combination, versus dynamic risk stratification using PVE. The ATA risk classification and postoperative s-Tg showed PVE values of 12.7% and 23.3%, respectively. When the two variables were combined, the PVE increased to 29.9% but was significantly lower than that observed with dynamic risk stratification (49.1%) (Table 5).

**Table 4:** Impact of response to treatment on initial risk estimates (dynamic risk stratification) in patients with papillary thyroid carcinoma

		Initial estimate of risk of recurrence	Dynamic risk stratification		
			Complete response	Indeterminate response	Incomplete response
ATA risk	Low	2.3% (21/932)	0.5% (4/806)	3.1% (3/97)	48.3% (14/29)
	Intermediate	9% (52/575)	1.2% (5/428)	14.6% (13/89)	58.6% (34/58)
	High	25% (26/104)	5.3% (3/57)	22.2% (6/27)	85% (17/20)
s-Tg (ng/mL)	<10	2.1% (28/1,312)	0.6% (7/1,153)	5.3% (7/132)	51.9% (14/27)
	≥10	23.7% (71/299)	3.6% (5/138)	18.5% (15/81)	63.8% (51/80)
ATA risk/postoperative s-Tg (ng/mL)	Low/<10	1.1% (9/815)	0.3% (2/736)	1.5% (1/69)	60% (6/10)
	Low/≥10	10.3% (12/117)	2.9% (2/70)	7.1% (2/28)	42.1% (8/19)
	Intermediate/<10	2.8% (12/433)	0.8% (3/368)	7.7% (4/52)	38.5% (5/13)
	Intermediate/≥10	28.2% (40/142)	3.3% (2/60)	24.3% (9/37)	64.4% (29/45)
	High/<10	11.1% (7/63)	4.2% (2/48)	18.2% (2/11)	75% (3/4)
	High/≥10	46.3% (19/41)	11.1% (1/9)	25% (4/16)	87.5% (14/16)

Abbreviations: ATA: American Thyroid Association; s-Tg: stimulated thyroglobulin.

**Table 5:** Proportion of variance explained obtained from logistic regression model of recurrence (Nagelkerke/Cragg-Uhler) evaluating the value of ATA risk stratification, postoperative stimulated thyroglobulin, and response to treatment

Variable	Category	n (%)	PVE	Odds ratio (95% CI)	P value
ATA risk	Low	932 (57.8)	0.127	1.0 (ref.)	
	Intermediate	575 (35.7)		4.31 (2.57-7.24)	<0.001
	High	104 (6.5)		14.46 (7.78-26.87)	<0.001
Postoperative s-Tg (ng/mL)	<10	1,311 (81.4)	0.233	1.0 (ref.)	
	≥10	300 (18.6)		14.21 (8.97-22.49)	<0.001
ATA risk/postoperative s-Tg (ng/mL)	Low/<10	815 (50.6)	0.299	1.0 (ref.)	
	Low/≥10	117 (7.3)		10.23 (4.21-24.87)	<0.001
	Intermediate/<10	433 (26.9)		2.55 (1.07-6.11)	0.035
	Intermediate/≥10	142 (8.8)		35.12 (16.56-74.49)	<0.001
	High/<10	63 (3.9)		11.19 (4.02-31.17)	<0.001
	High/≥10	41 (2.5)		77.34 (31.47-190.1)	<0.001
Response to treatment	Excellent	1,291 (80.1)	0.491	1.0 (ref.)	
	Indeterminate	213 (13.2)		12.28 (5.98-25.21)	<0.001
	Incomplete	107 (6.6)		164.95 (82.9-328.3)	<0.001

Abbreviations: ATA: American Thyroid Association; 95% CI: 95% confidence interval; ref.: reference; s-Tg: stimulated thyroglobulin.

## DISCUSSION

Studies analyzing PTC of all sizes have described recurrence rates ranging from 6.6% to 28% (3,18,19). In our series, we found a low recurrence rate (6.1%), which was probably influenced by some factors. First, we found a high percentage (54.7%) of papillary thyroid microcarcinomas (mPTCs). Additionally, most patients (57.8%) were asymptomatic and had ATA low-risk tumors. All these criteria are associated with a better prognosis (7,20,21). Indeed, according to some authors, an active surveillance management approach would be a safe and effective alternative to immediate surgical resection in these patients with low-risk PTC (22,23). In our cancer center, many mPTCs were discovered incidentally by neck imaging during follow-up for other malignancies or were found in surgical specimens in patients who underwent thyroidectomy for benign disease. Some patients were referred for thyroidectomy after ultrasound and fine needle aspiration of small tumors, a practice that has been discouraged in recent years. Second, all patients included in the present study had undergone total thyroidectomy, which may reduce local recurrence as it removes all potential cancer foci in both lobes (24). In fact, we found a multifocality rate of 40.3% (649 patients), with 471 patients (29.2%) having bilateral cancer foci. Third, most of the patients in our study received RAI, and some authors (25–27) state that the administration of RAI after thyroidectomy may be beneficial in reducing cancer recurrence in patients with low-risk to intermediate-risk PTC.

Several clinicopathological factors have been described in the literature as predictors of PTC recurrence (28). The ATA risk stratification system is a reliable predictor of tumor relapse in patients with differentiated thyroid carcinomas (DTC) and has been validated by different treatment centers (29–31), including ours (32). Many authors have confirmed the importance of obtaining postoperative s-Tg at the time of RAI administration. In general, high postoperative s-Tg levels (>1-2 ng/mL) are associated with a higher risk of recurrence (9,14,33), while even higher postoperative s-Tg levels (>10-50 ng/mL) are associated with distant metastases (34,35) and higher mortality (36,37) in patients with DTC. On ROC curve analysis, we found that the cutoff level of 10 ng/mL for postoperative s-Tg strikes the ideal balance between sensitivity and specificity in predicting PTC recurrence. This value is similar to the one found by Webb and cols. (9) in a meta-analysis including almost 4,000 patients treated for DTC across 15 centers. Using a postoperative s-Tg value < 10 ng/mL, these authors found a high negative predictive value (94.2%), which is comparable to the one observed by us (97.8%).

Measurement of postoperative s-Tg level may be important even in patients with low-risk to intermediate-risk PTC who undergo total thyroidectomy and do not receive RAI for remnant ablation, as low postoperative s-Tg values (<1 ng/mL) are associated with excellent results in these patients, with a reported recurrence rate below 1% (38). A small percentage of the patients in our cohort (12.7%, n = 204) did not receive RAI treatment. The majority of these patients had tumors with a good prognosis, *i.e.*, 187 (91.7%) had low-risk PTC, and 188 (92.2%) had postoperative s-Tg < 10 ng/mL. Among these patients, only 4 (2%) developed disease relapse; the recurrence in all these patients occurred in cervical lymph nodes, and their postoperative s-Tg levels ranged from 6 to 234 ng/mL. We agree that high postoperative s-Tg levels (>5-10 ng/mL) may be helpful in identifying patients with low-risk or intermediate-risk PTC who may benefit from RAI administration to improve staging or facilitate follow-up (5,7,39). In contrast, RAI administration may not be required in patients with PTC who demonstrate low post-surgical Tg levels, stimulated or not by TSH, in the absence of antithyroglobulin antibodies and with negative cervical ultrasound after thyroidectomy (40,41).

When measurement of postoperative s-Tg level was incorporated into the ATA risk assessment, an s-Tg level < 10 ng/mL was associated with a decreased risk of disease recurrence in all three ATA risk categories, i.e., from 2.3% to 1.1% in patients with low-risk, from 9% to 2.8% in those with intermediate-risk, and from 25% to 11.1% in those with high-risk PTC. In contrast, s-Tg levels ≥ 10 ng/mL were associated with an increased risk of disease recurrence in all three ATA risk categories, i.e., 10.3%, 28.2%, and 46.3%, respectively. Recently, Tian and cols., studying 2,524 patients treated for DTC, found results very similar to ours, demonstrating that the integration of postoperative s-Tg levels into ATA risk categories has an impact in modifying the initial risk estimates (42). Unlike these authors, we also incorporated data from dynamic risk stratification. Thus, patients with low to intermediate ATA risk and s-Tg levels < 10 ng/mL who demonstrated an excellent response to treatment (which corresponded to 1,104 cases or 68.5% of our cohort) had a very low recurrence rate (<0.8%). On the other hand, higher recurrence rates were observed in patients with intermediate to high risk and s-Tg ≥ 10 ng/mL who demonstrated an indeterminate response to treatment (24.3%-25%) and in those with incomplete response regardless of ATA risk category or postoperative s-Tg level (38.5%-87.5%).

According to the ATA guidelines published in 2015, postoperative RAI administration “is not routinely recommended” in low-risk patients, “should be considered” in intermediate-risk patients, and “is routinely recommended” in high-risk patients (7). However, considerable disagreement exists between authors regarding intermediate-risk patients, as demonstrated by Lamartina and cols. in a literature review (43). Available data suggest that the greatest potential benefit of RAI can be seen in patients of advanced age and in those with aggressive histologic variants (such as tall cell, diffuse sclerosing, and insular) or lymph node disease (bulky and/or outside the central neck compartment). In these intermediate-risk patients, the incorporation of postoperative s-Tg data may add valuable prognostic information supporting the decision to administer adjuvant RAI. Indeed, some authors have observed that delaying RAI treatment for more than 6 months after surgery has no impact on clinical outcomes (disease persistence) in patients with intermediate-risk DTC (44,45). This finding allows for a more relaxed attitude when planning for the selective use of RAI administration in patients with intermediate-risk PTC.

Some authors have shown PVE values significantly higher with dynamic risk stratification systems (62%-84%) compared with initial risk stratification systems (<30%) (8,46). Besides that, our results show that assessment of response to initial therapy was the most important variable, explaining 49.1% of the variance in disease recurrence, significantly higher than ATA risk (12.7%), postoperative s-Tg (23.3%), or the combination of these two variables (29.9%). These data indicate that long-term outcomes can be predicted more reliably using new information incorporated during follow-up.

The present study emphasizes the importance of incorporating postoperative s-Tg information into clinicopathological data for early prediction of both the pattern of response (biochemical and structural) to treatment and the risk of recurrence in patients with PTC. To our knowledge, this is the first study to assess the impact of dynamic risk stratification in refining initial risk estimates based on the ATA risk system in combination with postoperative s-Tg. We included an analysis of patients undergoing total thyroidectomy without RAI administration, showing data that suggest that dynamic risk assessment can also be applied to patients not treated with RAI.

Some limitations of this retrospective study are mainly related to selection bias. Many patients were excluded from the analysis due to insufficient information in medical records. Recommendations about treatment and frequency of follow-up visits and examinations varied from patient to patient based on individual surgeons and patient preferences rather than institutional protocol. This would lead to a higher diagnosis of recurrent disease in patients with intermediate to high risk due to more rigorous and frequent follow-up compared with low-risk patients. In addition, important prognostic variables included in the updated version of the ATA 2015 risk stratification system, such as the size of lymph node metastases, were not evaluated in this study. Another important limitation concerns the clinical applicability of the approach used in this study, since about two thirds of the patients included in the study had low-risk tumors and most were treated by total thyroidectomy followed by RAI ablation. In light of current knowledge, most low-risk (and some intermediate-risk) patients can be safely managed with total thyroidectomy without RAI administration, lobectomy alone, or active surveillance. Finally, a mean follow-up period of 61.5 months may be short, as some patients with less aggressive disease may experience clinically significant recurrence many years after initial therapy.

In conclusion, serum postoperative s-Tg level is an important prognostic tool, and its combination with the ATA staging system can provide early valuable information regarding the likelihood of response to therapy and disease control in patients with PTC. In addition, initial risk estimates can be significantly refined based on dynamic risk assessment following response to therapy, thus providing a useful guide for follow-up recommendations.
